# Examining the need for eye protection for coronavirus disease 2019 (COVID-19) prevention in the community

**DOI:** 10.1017/ice.2020.314

**Published:** 2020-06-24

**Authors:** Alexandre R. Marra, Michael B. Edmond, Saskia V. Popescu, Eli N. Perencevich

**Affiliations:** 1 Center for Access & Delivery Research and Evaluation (CADRE), Iowa City VA Health Care System, Iowa City, Iowa, United States; 2 Department of Internal Medicine, University of Iowa Hospital & Clinics, Iowa City, Iowa, United States; 3 Albert Einstein Jewish Institute for Education and Research, Hospital Israelita Albert Einstein, São Paulo, Brazil; 4 George Mason University, Fairfax, Virginia, United States

*To the Editor*—As the world reopens after extreme social distancing designed to flatten the curve and protect hospitals, it appears that even countries that had controlled coronavirus disease 2019 (COVID-19) with widespread testing and contact tracing, such as South Korea and Singapore, are seeing increased case counts. One proposed method for reducing transmission as society reopens is requiring the public to wear face coverings, including cotton face masks or face shields.^1^ An important factor that distinguishes face shields from masks is eye protection. Yet the importance of eye protection in the prevention of COVID-19 and other coronaviruses is underappreciated, which has led to public health authorities recommending cotton face masks over potentially more protective alternatives, such as face shields.

The mucous membranes of healthcare workers (HCWs), including the conjunctiva, may be exposed to respiratory droplets from the patient.^2^ The importance of eye protection during care of patients with novel coronaviruses was recognized in 2003 during the severe acute respiratory syndrome coronavirus (SARS-CoV-1) outbreaks and subsequent Middle East respiratory syndrome coronavirus (MERS-CoV) outbreaks.^3^ For example, during SARS, the lack of eye protection when transferring a patient may have been the primary risk factor for one of the first doctors infected.^4^

It has been increasingly recognized that severe acute respiratory coronavirus virus 2 (SARS-CoV-2) can be transmitted from infected individuals when they are asymptomatic or presymptomatic.^3,5^ Thus, to prevent transmission in the community, personal protective equipment (PPE) must be worn at all times in addition to other containment measures such as 2 m (6 feet) distancing and avoiding large gatherings. Both droplet and contact transmission routes have been implicated in the spread of SARS-CoV-2.^1,3^ PPE has 2 potential benefits when worn in the community: (1) PPE can provide source control by containing the respiratory droplets generated through coughs, sneezes or during speech and (2) PPE can act as a barrier preventing respiratory droplets from landing on facial mucosal membranes or other parts of the face. Additionally, PPE can prevent contact transmission by preventing contaminated hands from reaching the mucosal membranes of the mouth, nose and eyes.

Eye protection might provide additional benefits. A detailed investigation of risk factors for HCW acquisition of SARS, including multivariate generalized estimating equation logistic regression models, identified unprotected eye contact with body fluids as an independent risk factor for infection (odds ratio [OR], 7.34; *P* = .001).^6^ However, in a survey of 8 of the 9 US healthcare facilities in which SARS-CoV-1–infected patients were evaluated, 70% of HCWs reported some exposure to patients without wearing some level of eye protection and none acquired infection.^7^

Although conjunctivitis has been described in a few patients with COVID-19 and other coronavirus syndromes,^5^ emerging evidence supports that coronavirus can enter the host via the conjunctival route.^8^ Conjunctiva may be a potential portal for infection^9^ because it is directly exposed to extraocular pathogens, and the mucosa of the ocular surface and upper respiratory tract are connected by the nasolacrimal duct and have been shown to share the same entry receptors for some respiratory viruses,^5^ including angiotensin-converting enzyme 2 (ACE2) for SARS-CoV-1 and SARS-CoV-2.^2,5^ In addition, SARS-CoV-2 was detectable in several nasolacrimal system–associated tissues, including the conjunctiva, lacrimal gland, nasal cavity, and throat, thus validating the anatomical bridge between ocular mucosa and the respiratory tract.^8^ Finally, macaques were susceptable to SARS-CoV-2 infection via the conjunctival route and progressed to lung infections suggesting the biological importance of eye infection.^10^

Given that SARS-CoV-2 can be transmitted by fomites and droplets that contact the mucous membranes of the mouth and nose, as well as the eyes, it appears that until proven otherwise, HCWs and at-risk citizens in the community should use barriers to protect their entire face including their eyes. Current public health guidance recommends cotton face masks, but given the potential role of the conjunctival route, face shields that provide barrier protection for the entire face might be the superior option. Further research in this area is critically needed.
